# Cervical Posterior Instrumentation Surgery Using a Patient-Specific Screw Guide Templating System: A Case Report

**DOI:** 10.7759/cureus.47538

**Published:** 2023-10-23

**Authors:** Yukino Hirao, Hiroshi Takahashi, Masao Koda, Toru Funayama, Masashi Yamazaki

**Affiliations:** 1 Department of Orthopaedic Surgery, Institute of Medicine, University of Tsukuba, Tsukuba, JPN

**Keywords:** surgical assist device, cervical pedicle screw, ossification of the posterior longitudinal ligament, cervical compression myelopathy, cervical posterior instrumentation

## Abstract

Posterior cervical pedicle screw (CPS) is one of the most robust forms of posterior instrumentation. Nonetheless, the spinal cord, nerve roots, and vertebral artery are situated in proximity to the cervical pedicle, engendering a significant risk of damage to these structures during CPS insertion. Here, we report a case of cervical posterior instrumentation surgery using a patient-specific three-dimensional (3D) screw guide templating system approved for the cervical spine (Myspine Cervical®). A 62-year-old man presented to our hospital with progressive numbness and paresthesia in both hands and fingers, as well as gait disturbance, which had persisted for one year. A neurological examination revealed severe myelopathy. Imaging findings showed severe spinal cord compression due to ossification of the posterior longitudinal ligament (OPLL) from C3/4 to C6/7. On the diagnosis of compression myelopathy due to cervical OPLL, we performed a posterior decompression and fusion surgery using a patient-specific 3D screw guide templating system (Myspine Cervical®). No severe complications occurred during the surgery. Evaluation of the CPS position by postoperative CT showed that all the CPS placements were accurate. The implementation of the patient-specific 3D screw guide templating system facilitated the secure and precise insertion of CPS in comparison to other surgical assist devices.

## Introduction

Early surgery is the only treatment for compression myelopathy such as cervical spondylotic myelopathy and cervical ossification of the posterior longitudinal ligament (OPLL) [1]. Laminoplasty was developed as a posterior surgical procedure with favorable results. However, recent reports indicate that laminoplasty has a poor surgical outcome in some cases with local kyphosis and K-line minus OPLL, leading to the requirement of posterior decompression and fusion surgery (PDF) in such cases [2]. The cervical pedicle screw (CPS) is a robust posterior instrumentation developed by Abumi et al. [3] for numerous procedures of cervical posterior spinal instrumentation. However, there is a considerable risk of damage to the vertebral arteries, spinal cord, and nerve roots during CPS insertion because they are located around the cervical vertebra [4]. MySpine Cervical® (Medacta International SA, Casted San Pietro, CH) is a patient-specific three-dimensional (3D) screw guide templating system approved for the cervical spine [5]. Here, we report a case of cervical posterior instrumentation surgery using this screw guide system.

## Case presentation

**Figure 1:**
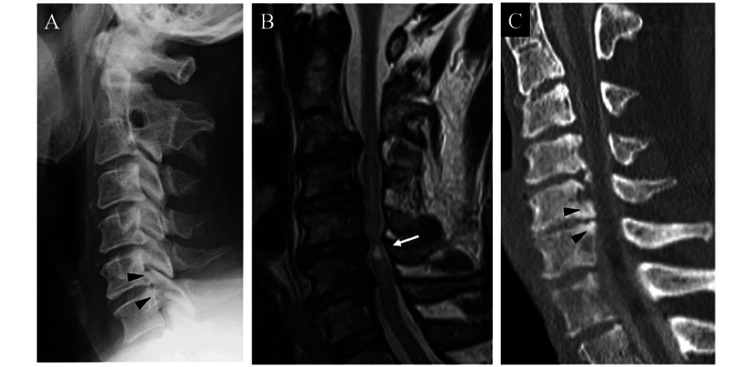
Imaging findings before surgery. A. Lateral X-ray image shows ossification of the posterior longitudinal ligament (arrowhead). B. MRI T2-weighted imaging shows a T2 high signal change at the C5/6 level (arrow). C. CT sagittal reconstruction image after myelogram shows ossification of the posterior longitudinal ligament (arrowhead).

A 62-year-old man presented to our hospital due to worsening numbness in both hands and fingers, abnormal sensation, and gait disturbance for the previous year. Neurological examination revealed numbness and abnormal sensation bilaterally in the C7 region, impaired fine motor control of both hands and fingers, gait disturbance, and increased deep tendon reflexes in both lower limbs. The Japanese Orthopaedic Association (JOA) score for cervical myelopathy was 10.5 out of 17 points. The X-ray revealed C2/3 fused vertebrae and segmental OPLL at the C5-6 levels (Figure 1A). MRI revealed spinal cord compression at C3/4, C5/6, and C6/7 accompanied by a T2 high signal change at C5/6 (Figure 1B). CT after myelogram revealed spinal cord compression due to segmental OPLL at the C5-6 levels (Figure 1C). We diagnosed the patient with compression myelopathy due to OPLL. In this case, the X-ray lateral image at a neutral position had a K-line (+). However, this case had a large range of motion at C4/5, and an X-ray lateral image at flexion showed a K-line (-). Thus, we performed a C2-7 PDF surgery using a patient-specific 3D screw guide templating system (MySpine Cervical®).

**Figure 2:**
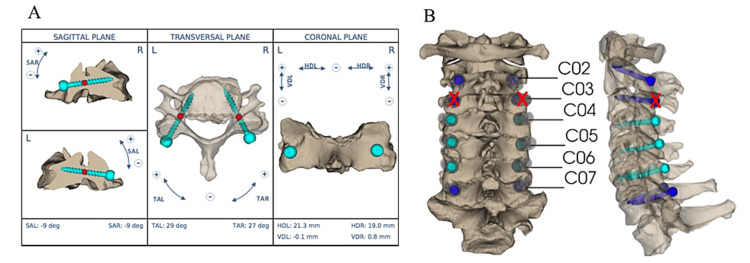
A. Preoperative planning for cervical pedicle screw (CPS) trajectory at the workstation. B. Three-dimensional imaging of CPS trajectory. CPSs were not inserted into C3 because the pedicle diameter was small.

**Figure 3:**
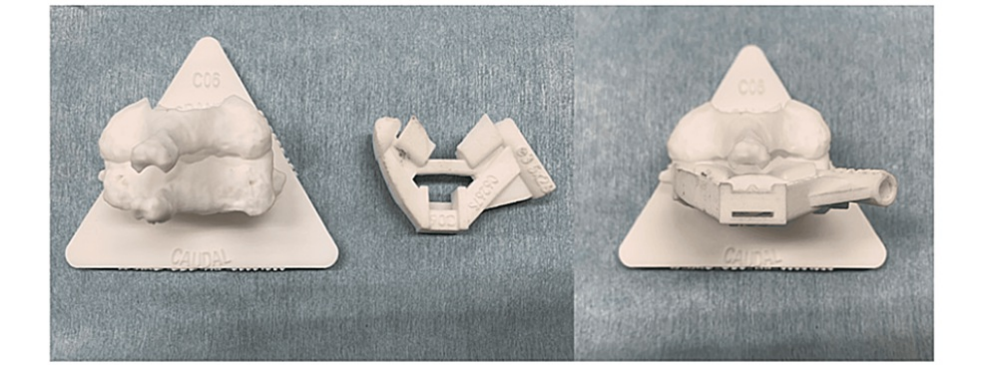
The three-dimensional (3D) bone model and a patient-specific 3D templating guide.

**Figure 4:**
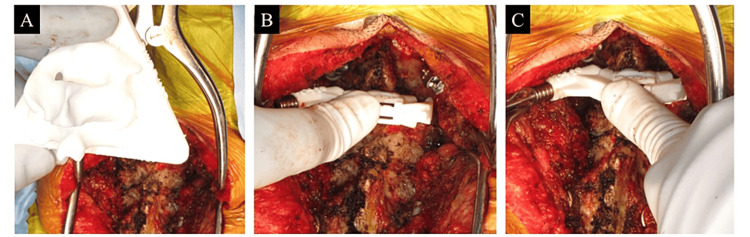
Intraoperative findings.

During preoperative planning, the trajectory of the CPS including the entry point, insertion angle, and screw length was simulated on a workstation and predetermined in advance for each vertebral body (Figure 2A). As a result of the simulation, we decided not to insert CPSs into C3 because the pedicle diameter was small, but planned to insert pedicle screws with 3.5 mm diameters made of titanium for C2 and C4-7 (Figure 2B). Based on the workstation, a 3D bone model and a patient-specific 3D templating guide made of reinforced polyamide were developed for each vertebra. We confirmed a good fit of the guide using the bone model (Figure 3). After autoclave sterilization of the bony model and templating guides, a C2-7 PDF surgery was performed (Figure 4). After lamina exposure, the bone morphology was compared carefully with the surgical field and the 3D model (Figure 4A), and osteophytes were removed. The guide was fitted firmly and stabilized using fingers to prevent the floating of the guide (Figure 4B). Drilling and tapping were then performed (Figure 4C), followed by the insertion of the pedicle screw. The C5 CPS insertion was aborted due to significant bone sclerosis observed during drilling. During surgery, a fluoroscopy lateral image was checked to confirm the CPSs were adequately inserted. Ultimately, we inserted a total of eight CPSs bilaterally into C2, C4, C6, and C7.

**Figure 5:**
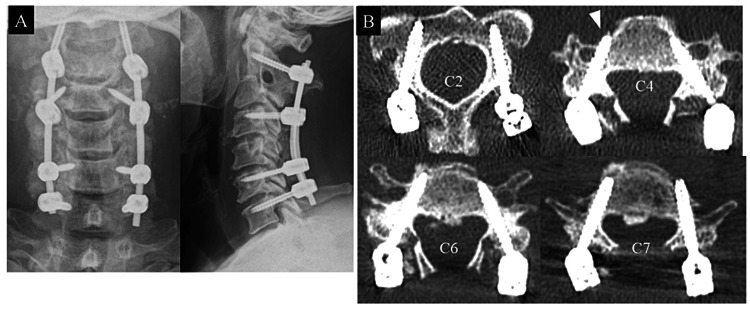
Postoperative imaging findings. A. Postoperative X-ray image. B. Postoperative CT axial image at each cervical pedicle screw (CPS) insertion level. The right side C4 CPS was Grade 1 in the Neo classification, and all the other screws were Grade 0.

After the decompression of the spinal cord with C3-6 double-door laminoplasty without using implants, the connection of rods was completed successfully. Finally, the local bone grafting to the lateral mass and facet of the fusion site using the removed spinous process from C3 to C6 was performed (Figure 5A). The Neo classification was used to assess the positioning of the CPSs by postoperative CT [4]. In the present case, the Neo classification showed that only one CPS was Grade 1 and the other seven CPSs were Grade 0, which indicated all pedicle screws were placed accurately (Figure 5B). Although postoperative transient paralysis in the left triceps due to intraoperative guide dislocation during C7 CPS insertion was observed, it recovered spontaneously three days after surgery. In addition, C5 palsy on the right side was observed four days after surgery. However, it showed spontaneous recovery at three months after surgery. The patient’s JOA score at the final follow-up one year postoperatively was 15 of 17 points, with a recovery rate of 69.2%, showing favorable improvement, although a long-term follow-up is required to monitor the long-term complications.

## Discussion

The perforation ratio for CPS insertion is from 6.7% to 27% under conventional fluoroscopy, creating a considerable risk of damage to the vertebral artery, spinal cord, and nerve roots [6-8]. To avoid this risk, various intraoperative support devices such as O-arm, navigation, robotic assistance, and patient-specific templating guides have been developed. To date, this system has been utilized to insert a total of 15 CPSs in three cases. Overall, 14 of 15 (93.3%) screws were Grade 0 in the Neo classification, with no deviations above Grade 2. Compared with other intraoperative-assisted CPS insertions, the patient-specific 3D templating guide system we applied had excellent results (Table 1). Although CPS insertion using O-arm navigation provides great accuracy for inserting CPS, the equipment is too expensive to be accessible in every hospital. In addition, it cannot reflect real-time changes in spinal alignment associated with drilling and screw placement [9,10]. Robotic assistance has developed only recently and is gradually gaining acceptance. However, there are still problems with insertion accuracy and surgical time [11]. Compared with other devices, the cost of a 3D bone model and a screw guide per single CPS is a list price of 20,000 JPY; therefore, the use of patient-specific 3D templating guides is relatively less expensive and provides great insertion accuracy similar to the past reports of using patient-specific guide system [12]. We are convinced that this system can be a good option for inserting CPSs.

**Table 1:** Accuracy of pedicle screw insertion using various intraoperative assistance.

Author	Number of pedicle screws	Neo classification			Levels of vertebra	Method of intraoperative assistance
		Grade 0	Grade 1	Grade 2 or 3		
Neo et al. (2005) [4]	86	61 (70.9%)	12 (14.0%)	13 (15.1%)	C2-C6	Fluoroscopy
Ishikawa et al. (2011) [9]	108	96 (88.9%)	9 (8.3%)	3 (2.8%)	C2-C7	O-arm navigation
Wada et al. (2020) [10]	317	305 (96.2%)	12 (3.8%)	0 (0%)	C2-C7	O-arm navigation
Farah et al. (2021) [11]	28	18 (64.3%)	6 (21.4%)	4 (14.3%)	C1-T3	Robotic assistance
Kaneyama et al. (2015) [12]	80	78 (97.5%)	2 (2.5%)	0 (0%)	C3-C7	Patient-specific 3D templating guide system
Fujita et al. (2021) [5]	77	76 (98.7%)	1 (1.3%)	0 (0%)	C3-7	Patient-specific 3D templating guide system
Our series	15	14 (93.3%)	1 (6.7%)	0 (0%)	C2-C7	Patient-specific 3D templating guide system

There are several pitfalls and limitations associated with the patient-specific 3D templating guide system. First, as in the present case, the dislocation of the guide can occur during intraoperative drilling, which may result in transient paralysis. When using this template, the guide must tightly fit into the vertebral arch, which requires adequate and careful posterior osteophyte and soft tissue dissection. Second, because it takes three weeks to create the guide system, it cannot be used for emergency cases such as trauma. Third, the number of cases is less, and comparative studies are not available. To evaluate the safety and feasibility of the procedure, further comparative and case-control studies are needed.

## Conclusions

The use of the MySpine Cervical®, a patient-specific 3D templating guide system for the cervical spine, allowed for safe CPS insertion with a high degree of accuracy compared with the other surgical assistive devices. Although there are some pitfalls and limitations, it is a good option when inserting the CPS.
